# Antibacterial-Based Hydrogel Coatings and Their Application in the Biomedical Field—A Review

**DOI:** 10.3390/jfb14050243

**Published:** 2023-04-25

**Authors:** Tai Peng, Qi Shi, Manlong Chen, Wenyi Yu, Tingting Yang

**Affiliations:** 1 Key Lab of Oral Biomedical Materials and Clinical Application of Heilongjiang Province, Jiamusi University, Jiamusi 154007, China; shiqi@jmsu.edu.cn (Q.S.);; 2 School of Materials Science and Engineering, Jiamusi University, Jiamusi 154007, China

**Keywords:** hydrogel coatings, antibacterial property, biomaterials

## Abstract

Hydrogels exhibit excellent moldability, biodegradability, biocompatibility, and extracellular matrix-like properties, which make them widely used in biomedical fields. Because of their unique three-dimensional crosslinked hydrophilic networks, hydrogels can encapsulate various materials, such as small molecules, polymers, and particles; this has become a hot research topic in the antibacterial field. The surface modification of biomaterials by using antibacterial hydrogels as coatings contributes to the biomaterial activity and offers wide prospects for development. A variety of surface chemical strategies have been developed to bind hydrogels to the substrate surface stably. We first introduce the preparation method for antibacterial coatings in this review, which includes surface-initiated graft crosslinking polymerization, anchoring the hydrogel coating to the substrate surface, and the LbL self-assembly technique to coat crosslinked hydrogels. Then, we summarize the applications of hydrogel coating in the biomedical antibacterial field. Hydrogel itself has certain antibacterial properties, but the antibacterial effect is not sufficient. In recent research, in order to optimize its antibacterial performance, the following three antibacterial strategies are mainly adopted: bacterial repellent and inhibition, contact surface killing of bacteria, and release of antibacterial agents. We systematically introduce the antibacterial mechanism of each strategy. The review aims to provide reference for the further development and application of hydrogel coatings.

## 1. Introduction

In the current biomedical field, medical devices, such as catheters, hernia nets, implants, and wound dressings, are often adhered by bacteria, leading to varying degrees of infection and posing a threat to the health of patients [1]. Once bacteria adhere to the surface of the substrate, they will rapidly form a biofilm, which attracts more bacteria, affecting the antibacterial effect of the immune system [2]. Therefore, the prevention of bacterial infection in the process of biomaterial implantation has become the focus of researchers. Surface coating or modification, which preserves the original properties of the material and changes only the surface properties, has been recognized as a promising strategy for introducing antibacterial efficacy into biomaterials. Some bionic surface morphologies with high aspect ratios are effective against colonization by bacteria, although the mechanism of antibacterial activity is not clear [3,4,5,6,7,8]. For methods of tailoring surface chemistry, substrates can be chemically modified or physically coated with a variety of antibacterial substances, including polymers, functional groups, inorganic nanoparticles, hydrogels, and antibiotics [9]. Among these bactericidal materials, hydrogel coating has many advantages and has been widely studied.

Hydrogels represent a polymer network system [10], i.e., a polymer with a three-dimensional network structure that can crosslink water molecules in the system by combining hydrophilic residues with water molecules. They can absorb a large amount of water, are swellable but do not dissolve in water, can maintain a specific shape, and have good elasticity [11]. Hydrogels can be fabricated from natural or synthetic polymers [12,13,14,15,16]. The advantages and disadvantages of some hydrogel raw materials are shown in Table 1. Hydrogels are very similar to natural active materials and are an ideal material for the preparation of medical dressings [17], drug delivery systems [18,19,20], and tissue engineering [21]. However, most hydrogels exhibit poor mechanical properties due to their uneven network structure and lack of energy dissipation mechanism, which lead to their limited application in the biomedical field. For example, hydrogels are easily deformed after repair of bone defects and thus need to be combined with other biomaterials or chemical modification to improve their performance. At present, biomaterials with hydrogel coating combine the excellent mechanical properties of implant devices and the biological properties of hydrogels, exhibiting excellent development prospects in the biomedical field [22]. Currently, the listed medical hydrogel coating products are mainly used in the catheters, stent delivery systems, and guide wires, among which the catheter accounts for the largest proportion, reaching 70% of sales in 2022. Brands include the Futae hydrogel coated latex catheter bag, the BIP catheter, and the Weili catheter.

The physical method of hydrogel coating to modify the surface of the biological substrate is simple, but it is not stable enough. The hydrogel coating is bonded to the surface of the substrate through a non-covalent bond, and the coating layer is easily delaminated from the surface [23,24]. Therefore, in order to improve the adhesion and stability of the hydrogel coating, it is necessary to use a chemical method (e.g., covalent bond) to attach the hydrogel coating [25,26]. Compared with other coatings (e.g., functional groups, self-assembled monolayers, and polymer brushes), hydrogel coatings exhibit many advantages. Firstly, hydrogel coatings have high grafting density and uniform coverage. Secondly, layer-by-layer (LbL) assembly coatings and polymer brush coatings are widely used, but the coating thickness has limitations. LbL hydrogel coatings are usually very thin, generally less than 100 nm, and they take much time to deposit layer by layer. The thickness of polymer brush coatings is related to the length of the polymer chain, generally less than several hundred nanometers [27]. In contrast, hydrogel coatings can be prepared by spin coating, electrochemical deposition, surface-initiated graft polymerization, and other methods, so the thickness of the hydrogel coating can be flexibly controlled from nanometer to micrometer, providing sufficient space to coat polymers, particles, small molecules, and other substances. Thirdly, unlike the non-crosslinked surface coating, which is attached with the substrate only by one covalent bond, the hydrogel coating is attached by multiple points. Therefore, hydrogel coatings are more stable for long-term modification.

We can classify antibacterial hydrogel coatings into two categories according to the type of active ingredients. The first is compositions containing antibacterial substances, such as natural or synthetic cationic polymers [28,29], amphoteric polymers [30], and antibacterial peptides [31]. The second is loaded antibacterial substances, such as antibiotics [32], antibacterial analogues [33], nano-silver [34], and zinc oxide, in the three-dimensional hydrogel network [35]. At present, researchers have conducted extensive research on the application of antibacterial hydrogels, but the research on hydrogels as a surface coating is not comprehensive. In this paper, the preparation methods for antibacterial hydrogel coating and its application in the biomedical antibacterial field are reviewed in detail.

## 2. Preparation Methods of Hydrogel Coatings

Hydrogels are prepared by chemical and physical crosslinking, but it is challenging to fix physically crosslinked hydrogels on the surface of materials due to the lack of binding sites for binding into the three-dimensional network [36,37]. In addition, the poor mechanical properties of physically crosslinked hydrogels compared to chemically crosslinked hydrogels limit the application of physically crosslinked hydrogels as durable coating materials [38]. The chemically crosslinked hydrogels can be gelatinized by monomer polymerization or conjugation reactions between polymer chains, whereby we can attach these hydrogels to the material surface in various ways to form stable hydrogel coatings [39]. The general strategies can be divided into three types: The first is surface-initiated graft crosslinking polymerization. The second is anchoring the hydrogel coating to the substrate surface. Third is the LbL self-assembly technique to coat crosslinked hydrogels [2].

### 2.1. Surface-Initiated Graft Crosslinking Polymerization

Free radical polymerization is a crucial way to prepare polymeric materials. The polymerization reaction is initiated by active radicals located on the surface of the substrate, so the adhesion of hydrogels can be promoted by generating active radicals on the substrate surface and introducing grafting sites on the surface [40].

#### 2.1.1. Direct Generation of Reactive Radicals on the Substrate Surface

**Figure 1:**
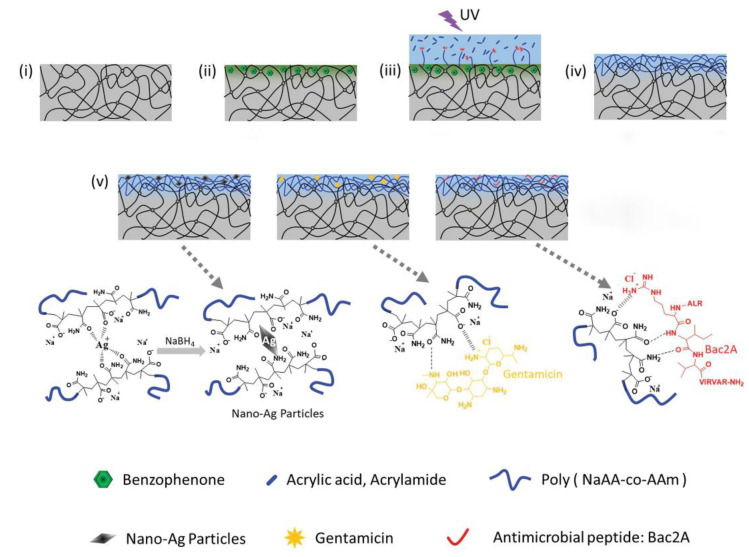
Preparation of the hydrogel coatings and antimicrobial agent loading [42]. (**i**) Pristine Polymer; (**ii**) Initiator Absorbing; (**iii**) In situ growth of hydrogel coating; (**iv**) As-formed hydrogel coating; (**v**) Antimicrobial agent loaded hydrogel coatings. Copyright 2021, The Royal Society of Chemistry.

Polymer materials are widely used in biomedical fields (e.g., heart valves, catheters, contact lenses). Mature strategies have been established for surface modification of inorganic materials, but general methods for polymer surface chemical modification are still lacking due to the relatively low surface inertness and surface energy of polymers. The photografting reaction may introduce surface-active radicals to grow polymer brushes and hydrogel coatings on the surface [41]. In a typical photografting process, the Norrish II type photoinitiator, e.g., benzophenone (BP), is excited to a singlet state (BPS) under irradiation by UV light and is then transformed to a triplet state (BPT) by intersystem crossing. It is well recognized that BPT is capable of abstracting hydrogen from other molecules or even solid substrates (RH), which generates reactive radicals (R•) and relatively less reactive benzopinacol radicals (BP-OH•). In the presence of vinyl monomer, R• acts as a reactive species to initiate radical polymerization, while BP-OH• is stabilized by the conjugate effect from the benzene ring and is not able to attack monomers but will couple with reactive radicals. Since polymer materials have plenty of C–H bonds, it is convenient to conduct photografting reactions on them. There have been some reports on the preparation of thin hydrogel layers by photografting on polymer substrates, and hydrogel coatings with antibacterial properties have also been investigated. For example, Zhang Fanjun et al. prepared a hydrogel antibacterial coating that can be applied to various blood-contacting devices (PVC and polyurethane pipes) (Figure 1) [42]. Free radical polymerization on the material surface was initiated with the photoinitiator benzophenone using an ultraviolet lamp. The dehydrogenation of benzophenone forms reactive radicals on the PVC polymer backbone and initiates monomer polymerization, resulting in a hybrid acrylamide and acrylic hydrogel coating. The inhibition zone test and confocal laser scanning microscopy revealed that the hydrogel coating could maintain remarkable antimicrobial and antifouling properties for four weeks. Furthermore, the hydrogel coating decreased the platelet adhesion/activation without risk of hemolysis. The ex vivo blood circulation study confirmed the antithrombotic properties of the hydrogel coating. Using impregnation methods to coat antibacterial drugs, Ag^+^, antibacterial peptides, and other substances may further improve the antibacterial properties of blood-contacting catheters. In another study, Fu Xiaoyi et al. prepared an ionic hydrogel coating doped with a Ni^2+^ trapping agent CS_2_ on the surface of a Nitinol bone implant through radical polymerization technology initiated by photografting, which may effectively be antibacterial. CS_2_ coated the released Ni^2+^ through chelating reaction to avoid cytotoxicity. This provides a research basis for embedding and detecting Ni^2+^ released from implantable biomaterials [43]. However, some polymers only have high-energy C(sp^2^)-H bonds, (e.g., polytetrafluoroethylene (PTFE), polyimide (PI)), which cannot be surface-modified by photo-grafting. Because the irradiation energy of traditional ultraviolet light is relatively low, these inert surfaces can be surface-modified by photo-grafting with excimer lasers.

#### 2.1.2. Introduction of Peroxide Groups on the Substrate Surface

**Figure 2:**
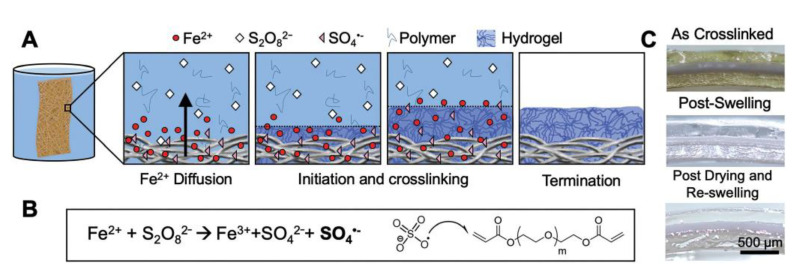
(**A**) Diffusion-mediated crosslinking, where Fe^2+^ diffuses away from the substrate into S_2_O_8_^2−^ to generate radicals and crosslink PEGDA hydrogels; (**B**) reaction between iron gluconate and the persulfate anion to generate sulfate radicals that initiate poly(ethylene glycol) diacrylate (PEGDA) end-groups; (**C**) hydrogel coatings immediately after crosslinking, after swelling for 24 h, and after drying and re-swelling [46]. Copyright 2020, The Royal Society of Chemistry.

Redox reactions between peroxide initiators and reducing agents may generate free radicals and initiate monomer polymerization. Peroxide groups may be activated under the irradiation of ultraviolet lamps, which initiates free radical polymerization. Therefore, the graft-crosslinking polymerization of hydrogels can be easily carried out on the surface, functionalized with peroxide groups. For example, Ma et al. prepared hydrogel coatings through a redox diffusion method in which Fe^2+^ mingled with the substrate material before 3D printing; when Fe^2+^ leached from the 3D printed structures, a redox reaction occurred with the persulfate ions S_2_O_8_^2−^ immersed in the solution, triggering the formation of crosslinked hydrogels [44,45]. However, Fe^2+^ leaching may have harmful effects on the properties of the substrate material; thus, Wancura Megan et al. developed a new method [46] in which the adsorption of Fe^2+^ onto the substrate would not have these effects. Iron (II) gluconate was adsorbed onto the substrate through a molecular adsorption method and immersed in a mixture of ammonium persulfate (APS) solution and polyethylene glycol diacrylate (PEGDA) to initiate a redox reaction between S_2_O_8_^2−^ and Fe^2+^ to produce sulfate anions and free radicals. Then, the sulfate radicals triggered the vinyl groups of PEGDA, initiating free radical crosslinking from the surface to produce a hydrogel (Figure 2). The prepared hydrogel coating has a controllable thickness and is formed through a gentle method that has proven to be effective in improving biocompatibility and preventing thrombus formation in subsequent experiments.

#### 2.1.3. Introduction of Catechol Groups on the Substrate Surface

Catechol groups can become functional on the surface of most organic or inorganic substrates [47]. The commonly used surface initiators of catechol groups include 3,4-dihydroxyphenylalanine and dopamine, which may form coatings with adhesive functionality on the surface of substrates through autoxidative polymerization mechanism. The generated quinones, amino groups, and phenolic hydroxyl groups can be used as reaction sites to combine with free radical polymerization initiators and promote graft crosslinking of hydrogels on the surface [48]. For example, in one study, Zhou et al. first deposited a layer of polydopamine on the surface of polydimethylsiloxane (PDMS) (American Braintree Scientific, Braintree, MA, USA) and then bonded the atom transfer radical polymerization (ATRP) initiator (α-bromoisobutyl bromide, BiBB was purchased from Sigma-Aldrich, Darmstadt, Germany) with hydroxyl and amino groups and fixed it to the surface of the catheter; the cationic monomer or crosslinker (polyethylene glycol dimethacrylate, PEGDMA was purchased from Sigma-Aldrich) initiated ATRP polymerization on the surface and formed an antibacterial hydrogel coating. The in vivo antibacterial and antibiofilm effect of these non-leachable covalently linked coatings (using a mouse catheter model) can be tuned to achieve a 1.95 log (98.88%) reduction and 1.26 log (94.51%) reduction of clinically relevant pathogenic bacteria (specifically with Methicillin-resistant Staphylococcus aureus (MRSA, were kindly provided by Kimberly Kline’s lab, which bought them from ATCC, Manassas, Virginia, USA) and Vancomycin-resistant Enterococcus faecalis (VRE)) [49]. This method of preparing antibacterial hydrogel coatings can also be used on small catheters with an inner diameter of 0.3 mm. In addition to radical polymerization, other methods can be used to prepare hydrogel coatings (e.g., Michael addition, Schiff-base addition, Diels-Alder addition). For example, Chen Yin et al. prepared an NOE hydrogel-coated stainless steel scaffold with nitric oxide elution [50]. The hydrogel precursor solution is directly coated on the surface of the scaffold. Due to the difference between melting point and body temperature, it is difficult to form a gel, so chemical crosslinking should be introduced. The study used the Michael addition reaction that promotes cytocompatibility. The previously used sulfhydryl-maleimide addition reaction was too fast and difficult to operate, so amine-maleimide addition was used instead of this reaction. Firstly, a P(DA-co-HDA) film layer was deposited on the stainless steel surface. The NOE hydrogel coating was prepared by coupling amine-maleimide and alginate, then crosslinking with gelatine and selenium species. The hydrogel coating can withstand balloon dilation, inhibiting excessive smooth muscle cell proliferation and preventing thrombus formation, and selenium species can also catalyze NO production and regulate cardiovascular homeostasis. Previous ISR prevention strategies rarely considered endothelial cell regeneration. This study increased endothelial cell adhesion and proliferation by grafting a hydrogel coating on the scaffold surface. The method has extensive research prospects.

**Figure 3:**
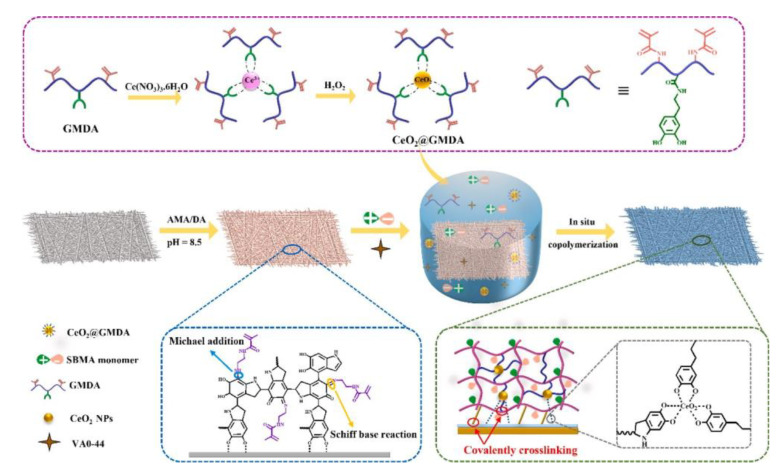
Schematic illustrations of preparation process of nanoceria-eluting degradable zwitterion hydrogel coating on PLLA membrane [53]. Copyright 2021, Chemical Engineering Journal.

Tugce et al. prepared a multifunctional “clickable” hydrogel coating through spin-coating [51], which enhanced the ability to move from protein fixation to cell attachment. Firstly, a dopamine methacrylamide layer was attached via catechol groups on the titanium surface. Then, furan-protected maleimide methacrylate (FuMaMA) hydrogels were prepared on the surface, and the furan groups were removed through a heating reaction, so that maleimide can react with sulfhydryl groups. Thus, the hydrogel can be functionalized by sulfhydryl maleimide nucleophilic addition and Diels-Alder cycloaddition reaction under mild conditions. The degree of functionalization of the hydrogel can be controlled by attachment of biotin-benzyltetrazine, followed by immobilization of TRITC-labelled ExtrAvidin, thus satisfying various biological properties. This method involves a simple synthetic principle and can couple various antibacterial substances, drugs, and biomolecules to achieve surface functionalization through various click reactions, allowing more diverse applications of hydrogel coatings. Liu prepared a polymer zwitterion coating based on poly(2-methacryloyloxyethylphosphorylcholine-co-dopamine methacrylate) (pMPCDA) copolymers with anti-inflammatory and antithrombotic properties [52], which are also used for blood-contacting catheters. In order to prepare such a uniform and stable coating, the PVC surface was amino functionalized by co-deposition of polydopamine (PDA) and polyvinyl imine. Then, based on various reaction mechanisms (e.g., Michael addition, Schiff-base addition) between catechol and amino groups, the zwitterionic pMPCDA copolymer was stably modified on the surface of PVC by using the mussel shell excitation chemical method. The hydrogel coating is not degradable, but degradable devices can reduce long-term complications in association with the residue of foreign materials in the body. Yang et al. prepared a membrane based on FDA-approved biodegradable Poly L-lactic acid (PLLA) material. First, dopamine (DA) groups are introduced on the surface of PLLA. Then, it is immersed in a synthetic Sulfobetaine methacrylate/Cerium oxide@Methacrylated gelatin dopamine conjugate (SBMA/CeO_2_@GMDA) solution. After Michael addition and Schiff base reaction, the nanoceria-eluting degradable zwitterion hydrogel coating is fabricated (Figure 3) [53].

#### 2.1.4. Introduction of Silane Coupling Agents on the Substrate Surface

Silane coupling agents may modify the surface of most inorganic or organic materials by introducing various functional groups as reaction sites, such as amine, aldehyde, thiol, vinyl, and quaternary ammonium groups [54]. Among them, the quaternary ammonium salt group has been proven by many researchers to have a very effective antibacterial ability. These reactive sites contribute to the adhesion of hydrogel coatings [55]. For example, Wu Xiaofang et al. studied an antibacterial hydrogel-coated artificial joint prosthesis [56]. In this study, the silane-coupling agent was loaded on the surface of laser-treated titanium alloy with hydrogen bonds; then, chitosan gelatin mixed hydrogel was prepared, which was immersed in nano-silver solution to form CS-GT-nAg antibacterial hydrogel. Using the 3D printing method, the hydrogel was connected with silane coupling agents by covalent bond and hydrogen bond, and firmly attached to the titanium alloy. The hydrogel coating has a dual-scale porous network structure, and the composite coating constructed by applying this method is more uniform and less prone to peeling, improving biocompatibility and antibacterial properties compared with porous coatings prepared by sol-gel [57], microarc oxidation [58], and laser sintering methods [59]. Refer to Table 2 for the above information.

### 2.2. Anchoring the Hydrogel Coating to the Substrate Surface

The preparation of stable polymer brushes on the material surface is possible by initiating graft-crosslinking polymerization on the substrate surface, which introduces a high density of reaction sites. However, the reaction efficiency is low, and the preparation of hydrogel coatings on the material surface does not require too many reaction sites. In general terms, the hydrogel can be thought of as a large molecule; immobilization on the material surface improves the coating binding stability and reaction efficiency.

#### 2.2.1. Click Chemistry for Anchoring Hydrogels to the Substrate Surface

Click chemistry has contributed significantly to the chemical synthesis field [60]. It has many applications, high yields, harmless byproducts, and simple reaction conditions. It is a widely used method in the field of biomedical research and includes the azide-alkyne cycloaddition reaction (AAC) [61], thiolene reaction [62], and Diels-Alder reaction [63]. The thiolene reaction is widely used for preparing hydrogel coatings due to its high reaction efficiency and fast gel formation. Magennis et al. functionalized polydimethylsiloxane (PDMS) with the silane coupling agent MTS [64]. Then, they used the introduced thiol groups as reaction sites to crosslink with multifunctional monomers to prepare hydrogel coatings immobilized on the substrate surface. The in vitro experiments showed effective bacterial growth inhibition compared to unmodified PDMS.

#### 2.2.2. Dopamine Group Functionalized Hydrogels Anchored on the Substrate Surface

Introducing dopamine groups into the hydrogel structure and anchoring on the substrate surface may avoid complicated pretreatment procedures on the substrate surface and improve the reaction efficiency.

**Figure 4:**
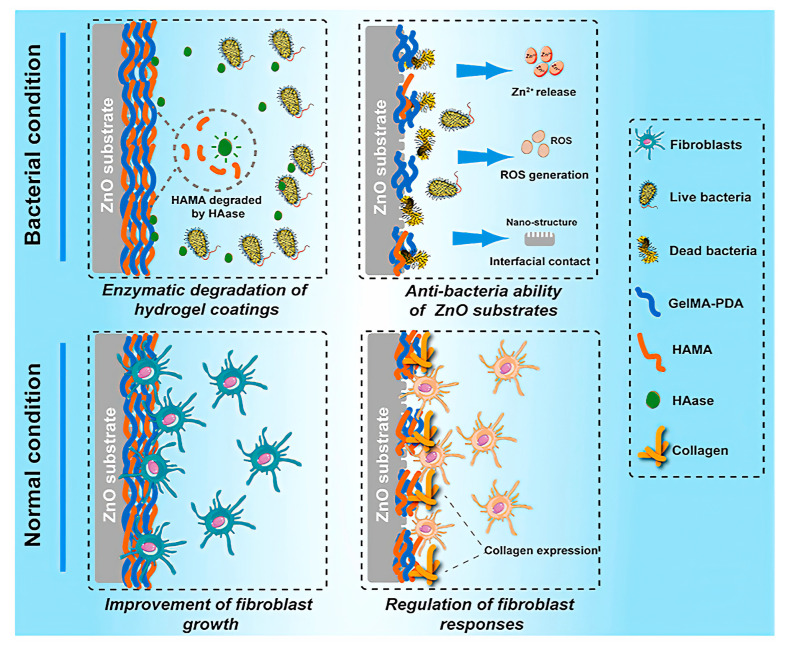
Schematic diagram of the self-adaptive strategy of [65]. Copyright 2021, Elsevier.

Leng Jin et al. constructed a ZnO layer with a nanoflower-like structure on a titanium surface by a hydrothermal method and then prepared a hybrid hydrogel of gelatine methacrylate (GelMA) and hyaluronic acid methacrylate (HAMA), which was firmly attached to the ZnO layer by grafting catechol motifs on the hydrogel and conducting photo-crosslinking (Figure 4) [65]. The composite could self-adapt. Under normal conditions, the hydrogel coating is stable and may effectively reduce the toxicity of ZnO. However, when bacterial infection occurs, the hydrogel coating can be effectively degraded by enzymes to release ZnO for antibacterial purposes, which further regulates the biological behavior of fibroblasts and exhibits good soft tissue compatibility while effectively balancing biosafety and antibacterial activity.

He Ye et al. used the same method to prepare catechol motif-modified gelatine methacrylate containing photosensitizer Chlorin e6-loaded mesoporous polydopamine nanoparticles (GelMAc/MPDA@Ce6), which were firmly attached to the titanium after 365 nm UV for 10 min [66]. Then, the photobiomodulation (PBM) method and photodynamic therapy (PDT) was performed by laser irradiation at specific wavelengths, which enabled the Ce6 photosensitizer to be activated to produce reactive oxygen species (ROS) and combine with other biomolecules to promote tissue repair. The coating exhibited effective antibacterial properties and promoted cell adhesion and proliferation. Combining PBM methods with hydrogel coatings of biomaterials to achieve biological functions is relatively novel and promising. Both studies used catechol moieties as anchoring groups to graft hydrogel coatings. The anchoring principle of catechol is that large amine groups, phenolic hydroxyls, and quinone structures formed by the oxidation of the derivative deposition layer may function as reactive sites to couple to initiators of active/controllable radical polymerization, which can be helpful in grafting antibacterial hydrogel coatings onto substrates. Catechol is an important pharmaceutical intermediate that can firmly attach a hydrogel coating to the substrate and is now widely used in biomaterials.

#### 2.2.3. Anchoring Hydrogel Layers by Free Radical Polymerization

The attachment of the hydrogel layer involves first prefixing the initiator on the substrate surface; then, the hydrogel precursor solution is polymerized on the substrate surface. In addition, instead of initiating polymerization on the surface, the hydrogel layer may initiate polymerization in the precursor solution and then be chemically anchored to the substrate surface through grafting groups on the surface of the hydrogel layer.

**Figure 5:**
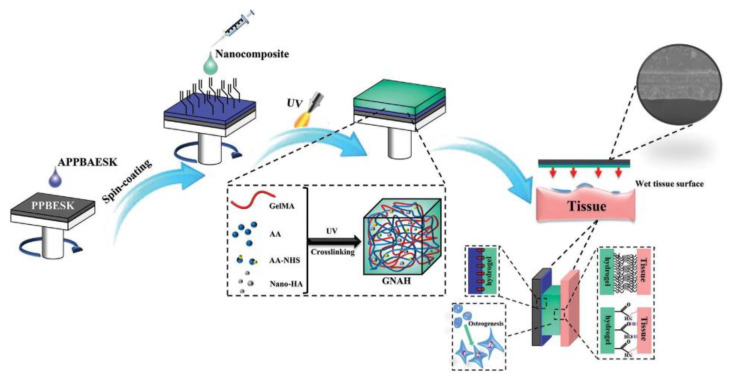
Preparation process of the chemically anchored bifunctional nanocomposite hydrogel coating. Reproduced with permission of [67]. Copyright 2021, John Wiley and Sons.

Liu Chengde et al. prepared a bifunctional nanocomposite hydrogel coating on the surface of poly(aryl ether ketone) PAEK implants [67,68]. The raw materials for hydrogel preparation included type A gelatine, acrylic acid (AA), N-succinimidyl acrylate (AAc-NHS ester), nano-hydroxyapatite, and methacrylic anhydride (MA). As shown in Figure 5, bulk nanocomposite hydrogels were first prepared by dissolving AA, GelMA, AAc-NHS ester, and α-ketoglutaric acid (KGA) in deionized water, then mixed with nanohydroxyapatite in different proportions and cured in an ultraviolet light (UV) chamber for 15 min [69,70]. A novel poly (phthalazinone ether sulfone ketone) containing allyl groups (APPBAESK) was spin-coated on the surface of PPBESK. Then, the already prepared bulk hydrogel was spin-coated on the surface and crosslinked under UV irradiation to form the coating. NHS-ester activated groups and nano-HA were introduced to endow tissue adhesivity and osteogenic activity. Chemically inert PPBESK was successfully functionalized by spin-coating of APPBAESK containing allyl groups. The nanocomposite hydrogel coating containing nano-HA and NHS-ester activated groups was chemically anchored on the surface of modified PPBESK, which greatly improved its hydrophilicity. The resulting bifunctional PPBESK could adhere to tissues quickly and with high adhesion strength up to 300 KPa in pig skin. In addition, osteoblasts and fibroblasts could adhere and proliferate well on the surface of nanocomposite hydrogel coatings on PPBESK. Importantly, ALP expression and osteoblastic differentiation of preosteoblasts cultured with modified PPBESK were promoted by the nanocomposite hydrogel coating containing nano-HA. In conclusion, this research provides a new modification strategy of PAEK implants for integrating osteogenic activity and tissue adhesivity. Refer to Table 3 for the above information.

### 2.3. LbL Self-Assembly Technique to Coat Crosslinked Hydrogels

The layer-by-layer self-assembly technique may prepare coatings on substrate surfaces through alternating deposition methods [71], and the coatings can be attached mainly by hydrogen bonding, electrostatic interactions, and charge transfer interactions. Because of the low stability of such noncovalent bonding forces, each layer can be deposited conjugately by covalent bonding in the preparation of hydrogel coatings. The LbL self-assembly technique is more suitable for preparing ultrathin hydrogel coatings. The substrate surface needs pretreatment, and the covalent attachment of the layer to the substrate surface may be realized by grafting functional groups. For example, the surface of the silicon wafer undergoes functionalization through reactants containing dopamine groups [72]; an ultrathin hydrogel coating can be prepared by depositing alternating polyacrylic acid (PAA) and chitosan quaternary ammonium salts using the LbL self-assembly technique. The coating surface is positively charged and effectively inhibits bacterial adhesion.

**Figure 6:**
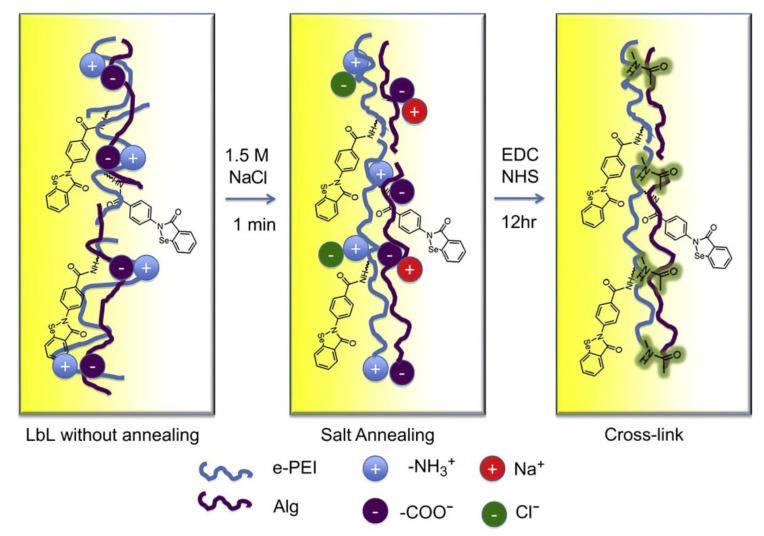
Schematic illustration of two-step annealing of LbL films, including salt annealing and cross-linking [73]. Copyright 2011, Elsevier.

Cai et al. prepared polyethylenimine (e-PEI) and alginate (Alg) conjugated with carboxyl-ebselen using the LbL self-assembly technique and then prepared multilayer films by crosslinking with the coupling agent EDC (Figure 6) [73]. Moreover, the functional membrane generated by the reaction of selenate with oxygen may produce superoxide, which inhibits bacterial adhesion. When (e-PEI/Alg)50 was placed in 105 CFU/mL *E. coli* at 37.5 °C, it was found that all the bacteria were killed after 24 h. The hydrogel coating may kill broad-spectrum bacteria.

Wang et al. synthesized Poly[oligo(ethylene glycol)fumarate]-co-poly[dodecyl bis(2-hydroxyethyl)methylammonium fumarate] (POEGDMAM) containing multi-enes and poly[oligo(ethylene glycol)mercaptosuccinate] (POEGMS) containing multi-thiols by polycondensation reaction, and the two synthesized functional polymers were deposited on thiosilicate substrates by the LbL self-assembly technique and “click” chemistry to prepare a crosslinked hydrogel coating [74]. The antibacterial activity of the ultrathin hydrogel films on silicon wafers was determined by the disc diffusion method against Gram-negative (Escherichia coli) and Gram-positive (*Staphylococcus aureus*) bacteria. The control groups without any hydrogel films generated no inhibition zones, while the others exhibited inhibition zones for both of these types of bacteria. The zones were quite obvious, considering the thickness of the films was just below 100 nm. Moreover, the inhibition zones increased significantly with the number of bilayers, which confirmed the LbL reaction on the substrates. Refer to Table 4 for the above information.

In summary, the above three preparation strategies have their advantages. The tolerance of surface-initiated graft crosslinking polymerization to water and impurities as well as its compatibility with various functional monomers enable the hydrogel coating to be effectively loaded on the surface of the substrate; the photograft crosslinking method can achieve the regulation of the overall or local antibacterial properties of the material. Photo- driven polymerization can be carried out in vivo in a non-invasive way, which has a good biomedical application prospect. Anchoring the hydrogel coating to the substrate surface is simple and does not require too many reaction sites, and the entire composite has a “sandwich” structure, which has a positive impact on the subsequent antibacterial modification. The LbL self-assembly technique to coat crosslinked hydrogels has long-term stability and is suitable for use as a carrier for slow release of antibacterial drugs. The preparation process is simple, gentle, and fast, and the hydrogel coating can act on the human body for a long time. Researchers can design different grafts and functional hydrogel coatings according to their needs.

Thus far, this review has detailed methods for preparing hydrogel coatings on the substrate surface of biological materials. At present, it is necessary for biomedical materials to have effective antibacterial properties. Although hydrogel itself has certain antibacterial properties, the antibacterial effect of being implanted into human body as a coating is not sufficient; thus, it is necessary to combine antibacterial substances with hydrogel coatings through reasonable chemistry to enhance the antibacterial effect. Next, we will introduce the antibacterial mechanism of hydrogel coating by classification.

## 3. Hydrogel Coatings in Biomedical Antibacterial Applications

**Figure 7:**
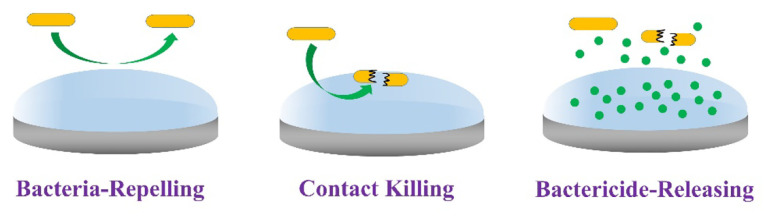
Strategies for antibacterial hydrogel coatings [2]. Copyright 2020, Elsevier.

The following three antibacterial methods are the main methods for material surface modification by hydrogel coatings: The first is bacterial repellence and inhibition. The second is the contact surface killing of bacteria. The third is the release of antibacterial agents (Figure 7) [2].

### 3.1. Bacterial Repellence and Inhibition

In the earliest stage of bacterial biofilm formation, bacterial adhesion on the surface is reversible, so the introduction of hydrogel coatings that repell bacterial adhesion is the most direct antibacterial method. The hydrogel coating prepared by this method has better biocompatibility [75]. When biomaterials are implanted into the body, proteins tend to absorb nonspecifically on the surface, which promotes bacterial adhesion; thus, the repellent hydrogel coating should effectively inhibit the nonspecific absorption of proteins. Substances with hydrogen bonding acceptors and hydrophilic polar functional groups may inhibit the nonspecific absorption of proteins; they may form hydrogen bonds with water molecules in aqueous media and form a highly hydrated layer on the polymer surface to effectively achieve antibacterial properties. Polyethylene glycols (PEG) [76], polyvinyl alcohols (PVA) [77], polyacrylates [78], amphiphilic polymers [79], polysaccharides, and other hydrophilic substances are widely used as raw materials in the field of antibacterial hydrogels [80]. Next, the applications of hydrogel coatings containing the above substances in biomaterials are introduced.

PEG has excellent biocompatibility. The easiest way to prepare PEG hydrogels is to crosslink the PEG derivatives with two capped vinyl groups, such as polyethylene glycol diacrylate (PEGDA), or copolymerize the PEG macromonomer containing one vinyl group at the chain end, such as (PEGMA). Ekblad and his colleagues prepared PEGMA copolymer hydrogel coatings on the surface of biomaterials, reducing the sedimentation density of bacteria to less than 5% compared to that on the original surface [81]. Johnbosco Castro et al. used a spray coating technique to uniformly distribute a reactive hydrogel precursor on cobaltchromium (CoCr) vascular stents and left the solution polymerizing to form a hydrogel coating [82]. The hydrogel coating is a mixture of four-armed PEG and heparin with disilane and poly (ethylene-alt-maleic anhydride) (PEMA) as a bonding layer, ensuring that the hydrogel coating can provide covalent immobilization on the stent surface. Bioassay results showed that the PEG-based hydrogel coatings could effectively prevent the settlement and accumulation of bacteria.

PVA hydrogels can be prepared by physical and chemical crosslinking, have excellent biocompatibility and hydrophilicity, and are widely used in the field of biomaterials. Li et al. applied polyurethane prepolymer/polyvinyl alcohol (PPU/PVA) hydrogel coatings on the surfaces of polydimethylsiloxane (PDMS) nerve electrodes and compared these with the surfaces of nerve electrodes not coated with any substance and only coated with a polyurethane prepolymer (PPU). The surface nonspecific fibrinogen adsorption was reduced by 92%, proving that polyvinyl alcohols have effective antibacterial properties [83]. Yan et al. embedded quaternized chitosan-coated molybdenum disulfide (QCS-MoS_2_) nanomaterials in PVA hydrogels. QCS-MoS_2_ has photoresponsive properties that improve the mechanical strength of PVA hydrogels; it also has good photothermal conversion ability to generate reactive oxygen species under the irradiation of near-infrared light at 808 nm, which enables the hydrogels to exhibit excellent antibacterial activity while remaining non-cytotoxic to L929 cells [84].

**Figure 8:**
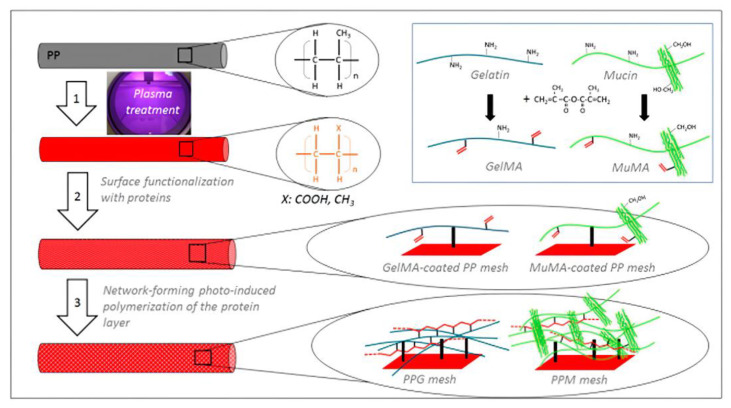
Coating characterization: Schematic representation of the three-step procedure used for the surface functionalization of PP meshes: 1—plasma treatment, 2—functionalization with protein analogues GelMA and MuMA (synthesis in inset), 3—generation of polymer-based hydrogels as coatings for the PP fibers. Reproduced with permission of [92]. Copyright 2020, MDPI polymers.

Polyacrylate hydrogels may form free radical polymerizations initiated by carbon—carbon double bond functional groups. Polyester blocks may hydrolytically degrade these hydrogels after implantation as a surface coating for biomaterials. The degradation products eventually exit the body through internal circulation. Every year, millions of repair procedures occur in hospitals worldwide, and inguinal hernia repair is one of the most common procedures [85,86]. In the 1960s, polypropylene (PP) mesh emerged as a biomaterial for hernia repair. In addition to PP, polytetrafluoroethylene (PTFE), polyurethane (PU), and polyethylene (PE) can also be used for hernia repair [87,88,89,90,91]. Since a synthetic mesh implanted in the human body produces various adverse reactions in the physiological environment due to the presence of bacteria, leading to chronic pain and discomfort, a polyacrylate hydrogel coating was introduced to avoid bacterial adhesion on the implant surface while demonstrating antibacterial properties and promoting cell proliferation. For example, Andrada Serafim et al. prepared GelMA and MuMa hydrogel coatings on the surface of a PP mesh by EDC-NHS chemical grafting (Figure 8) [92]. The study showed that the GelMA hydrogel-coated scaffold interacted most strongly with fibroblasts, and this interaction was further enhanced when GelMA was combined with PRP, indicating that the coating could promote wound healing. The coating effectively prevents the adhesion of Gram-positive and Gram-negative bacteria, opening up a novel direction for the application of bioactive meshes for ventral hernia repair.

Polyacrylamide (PAM)-based hydrogel coatings have more antibacterial activity than polyacrylate coatings because of the higher surface hydration capacity of the amide groups. PAM-based hydrogels are polymers consisting of acrylamide (AAM) copolymerized with other monomers and are widely used in biomedical antibacterial materials. For example, Zhang J. et al. prepared a hydrogel by combining chitosan and poly(N-(2-hydroxyethyl)acrylamide) (PHEAA), which exhibited excellent mechanical properties with a fracture stress of 3.8 MPa and a strain of 640%. The antibacterial property of PHEAA may effectively inhibit the adsorption of nonspecific proteins, endowing the hydrogel excellent antibacterial properties [93].

We can combine the above substances in two or more reasonable combinations to obtain better antibacterial properties. Although the bacteria-repelling strategy has a good effect, it cannot kill pathogens in body fluids. Moreover, PEG-based coatings and other polymer coatings cannot completely prevent the adhesion of bacteria.

### 3.2. Contact Surface Killing of Bacteria

Researches show that the combination of bactericidal compounds and hydrogel coatings can effectively kill bacteria. Different from the passive antibacterial mechanism of bacterial-repelling hydrogel coatings, the bactericidal hydrogel coating can actively kill bacteria by destroying the cell membrane of bacteria, thus preventing the propagation of bacteria and achieving effective antibacterial activity. The bactericidal compounds commonly used generally contain cations and hydrophobic groups, and since bacteria have negative charges, they can be adsorbed by the cations of the bactericidal compounds. The hydrophobic groups of bactericides may also damage the lipid composition of the bacterial membranes. Some widely used bactericides are antimicrobial peptides (AMPs) and quaternary ammonium compounds (QACs) [94,95].

**Figure 9:**
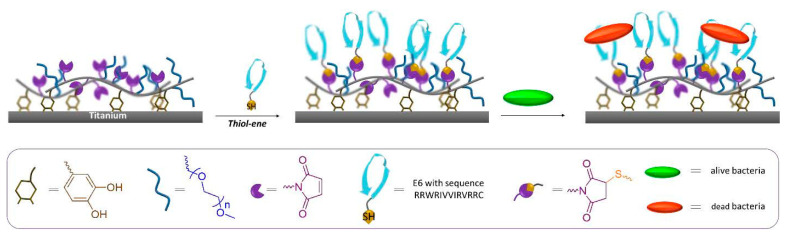
Schematic diagram of polymer hydrogel coating coupled with antimicrobial peptides to enhance the antibacterial performance. Reproduced with permission of [96]. Copyright 2019, American Chemical Society.

Antimicrobial peptides (AMPs), also known as host defense peptides, can be produced by plants, animals, humans, and bacteria. AMPs have effective antibacterial activity as the first line of defense against pathogenic invasion. The active bactericidal components of AMPs are arginine and lysine cations as well as a high proportion of hydrophobic amino acids. The antibacterial mechanism of AMPs is similar to that of quaternary ammonium compounds, which damage bacterial membranes through electrostatic interactions and hydrophobic groups. The AMPs may also self-assemble into physically crosslinked hydrogels with practical antibacterial ability. Tugce et al. anchored polymer films containing maleimide groups on the surface of titanium with catechol and then coupled them with AMPs by click chemistry. The antibacterial activity was enhanced, with an antibacterial rate of up to 80% (Figure 9) [96].

The bactericidal mechanism of quaternary ammonium compounds is the hydrophobic binding between cations and protein molecules, which adhere to bacteria, aggregate on the cell wall, and produce a chamber resistance effect to kill bacteria. In addition, the hydrophobic groups of QACs may interact with the hydrophilic groups of bacteria, changing the permeability of bacterial membranes and damaging the bacterial membrane structure. Combining QACs with hydrogel coatings may effectively confer antibacterial ability to the coatings and kill bacteria on contact, reaching nearly 100% antibacterial effect against Gram-positive and Gram-negative bacteria. Tao et al. prepared a hydrogel coating based on disulfide bonds by initiating a photopolymerization reaction of N-hydroxyethyl acrylamide (HEMAA), methylacryloxyethyl trimethyl ammonium chloride (DMC), and bis(2-methylpropene) ethoxydisulfide (DSDMA) on the surface. The coating combines the anti-adhesion property of poly(N-hydroxyethyl acrylamide) (PHEAA) with the bactericidal property of poly(quaternary ammonium salt), effectively inhibiting the adhesion and infection of E. coli and marine Vibrio. Ren et al. grafted QACs onto a polyurethane (PU) surface via surface-initiated atom transfer radical polymerization (SI-ATRP), and then grafted PVP hydrogel onto the surface by a Fenton-like reaction. QACs could improve the hydrophilicity of PU, and the surface water contact angle decreased from 93.6° to 60°, inhibiting bacterial adhesion and killing bacteria [97].

**Figure 10:**
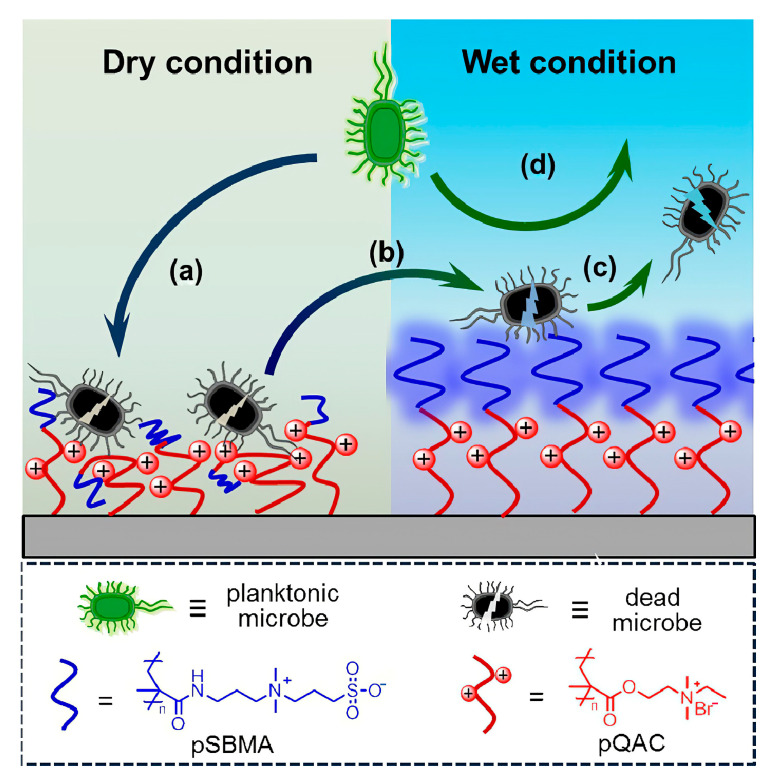
Antibacterial Surface in Dry and Wet Environment. In the dry state, the zwitterionic outer layer collapses, and the polycationic antibacterial layer kills bacteria (**a**); the collapsed zwitterionic outer layer swells (**b**) and allows the release of dead bacteria in the wet state (**c**). In a wet environment, the zwitterionic outer layer also prevents bacterial adhesion (**d**). Reproduced with permission of [98]. Copyright 2016, American Chemical Society.

AMPs and QACs have excellent bactericidal properties but cannot distinguish between normal cells and bacteria, resulting in poor biocompatibility. A combination of repellent and bactericidal mechanisms have been used to overcome these drawbacks. For example, Yan et al. prepared a composite coating of poly (N, N-dimethyl aminoethyl methacrylate) block copolymer (PDMAEMA) and PSBMA using a surface-initiated photoiniferter-mediated polymerization (SI-PIMP) strategy. PSBMA formed a zwitterionic outer layer on the PDMAEMA layer. In the wet state, it can not only achieve the effect of repelling bacteria, but also reduces the erosion of normal cells on the cationic part. In the dry state, the amphoteric outer layer collapses to expose the cationic part, achieving a bactericidal effect. The method enhances the biocompatibility of the composite (Figure 10) [98].

**Figure 11:**
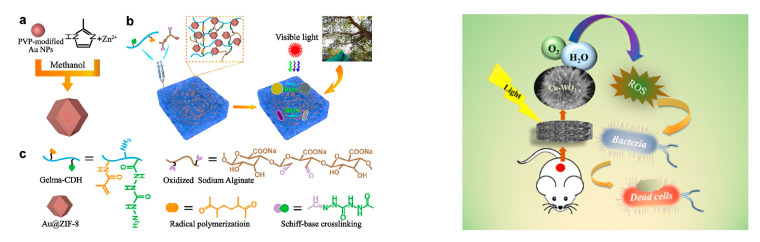
On the left is a diagram of reference [99]. (**a**) Schematic representation of Au@ZIF-8 synthsis; (**b**) Graphic illustration of Au@ZIF-8 imbedded into the hydrogels for light-driven treatment of the infection model; (**c**) Representaive chemical structures of individual components of the hydrogels. Copyright 2021, Elsevier. On the right is a diagram of reference [100]. Copyright 2023, American Chemical Society. The bactericidal mechanism of the two images is the same. Both produce reactive oxygen species (ROS) through photocatalytic semiconductor composite hydrogels to achieve bactericidal purposes.

Photocatalytic semiconductor composite hydrogels with reactive oxygen species (ROS)-generating capability have also attracted great attention for their contact bactericidal performance. Currently, such composite hydrogels are widely studied in the field of biological dressing coatings. Deng et al. prepared a hydrogel using oxidized sodium alginate and carbohydrazide-modified methacrylated gelatin as the matrix. Au@ZIF-8 semiconductor nanomaterials with MOF structure were embedded in the hydrogel. Au@ZIF-8 had the capability of photocatalysis, which can kill bacteria in contact with the wound surface by producing ROS under the irradiation of visible light [99]. Xing et al. prepared a chitosan sponge hydrogel coated with copper doped WO_3-x_ semiconductor material, which also had the same bactericidal mechanism (Figure 11) [100].

### 3.3. Release of Antibacterial Agents

The three-dimensional network structure of hydrogels can be loaded with antibiotics, AMPs, cationic polymers, silver ions, copper ions, antibacterial drugs, and other bactericidal compounds to enhance the antibacterial properties of the biomaterials. Compared with the above two methods, this method is more flexible and controllable, repelling bacteria and killing bacteria via released antibacterial agents. However, the preparation process is relatively complex and requires consideration of the release rate and concentration of the antimicrobial agents. For example, Hoque et al. prepared a biocompatible hydrogel using dextran methacrylate (Dex-MA) as a monomer and encapsulated it with a small molecular cationic biocide by in situ loading during photopolymerization. The hydrogels showed a sustained release of biocide and displayed 100% activity against methicillin-resistant Staphylococcus aureus (MRSA) for an extended period of time (until day 5) [101].

Due to the limitation of the surface hydrogel coating function, the coated antibacterial substance will become depleted gradually and cannot maintain an excellent antibacterial effect for a long period. Therefore, the release rate of antibacterial substances should be reasonably controlled. Thus, an intelligently controlled release coating structure can be prepared to release antibacterial substances under specific conditions (pH, light reaction, temperature, and REDOX reaction) to kill bacteria and free bacteria attached to the material surface. The applications of these intelligent controlled-release hydrogel coatings will be described in the following sections.

Yan Kun et al. prepared composite hydrogel antibacterial coatings on stainless steel needle electrodes via a two-step electrochemical strategy [102]. First, electrochemical deposition was conducted to combine chitosan and other monomers on the surface of the stainless steel needle electrodes to form a hydrogel coating. Second, silver nanoparticles were synthesized in the hydrogel coating through an in situ electrochemical synthesis method. The hydrogel coating has a layered structure and releases silver nanoparticles in stages through the change in pH value to achieve controllable antibacterial properties. This method is environmentally friendly and promotes the development of multifunctional nanomaterials in the biomedical field.

Xue et al. prepared a Dexp-loaded CuS nanoparticle cross-linked PEG hydrogel coating on the surface of 3D-printed polycaprolactone PCL scaffolds [103]. First, Dexp was loaded onto CuS NPs; then, the D-CuS-PEG hydrogel was prepared by crosslinking in PEG polymer, and the coating was formed on the surface of the PCL scaffold. CUS NPs had excellent photothermal conversion capability and high drug-loading capability. They could release Cu^2+^ and dexamethasone sodium phosphate under 1064 nm NIR irradiation and had excellent antibacterial activity.

Chandna et al. prepared a lignin-based hydrogel with both pH and light response properties; the hydrogels were doped with a photosensitizer (Rose Bengal, RB) and also with RB-conjugated lignin-derived silver nanocomplexes (RB@L-AgNCs). The RB@L-AgNCs were released under acidic conditions and irradiated with a green laser for 3 min. Reactive oxygen species (ROS) were produced, which greatly reduced the survival rate of bacteria. In subsequent studies, the hydrogel could be used in wound dressings and nanocoatings to achieve a stimulus-responsive antibacterial effect [104].

Li et al. loaded simvastatin into titanium dioxide nanotubes and prepared a thermosensitive chitosan-glycerin-hydroxypropyl methyl cellulose hydrogel (CGHH) coating on the surface of the nanotubes. At a normal body temperature of 37 °C, the CGHH coating was in the sol state, which resulted in the controlled release of simvastatin and promoted the differentiation of osteoblast *MC3T3-E1*. During bacterial infection, the CGHH coating transitioned into a gel state as the temperature rose to 40 °C, releasing glycerol and inducing macrophage polarization to the pro-inflammatory M1 phenotype to kill the bacteria [105].

Han et al. prepared a C-HA-Cys-allicin hydrogel coating with catechol-modified hyaluronic acid, cysteine, and allicin, and placed the hydrogel coating in an H_2_O_2_ solution. They found that the coating could be oxidized, which proved its REDOX response performance. Then, rhodamine was loaded into the hydrogel coating. It was found that the coating could release the drug in a REDOX environment for antibacterial purposes, and the best antibacterial effect occurred at a drug concentration of 5 μg/mL [106].

**Figure 12:**
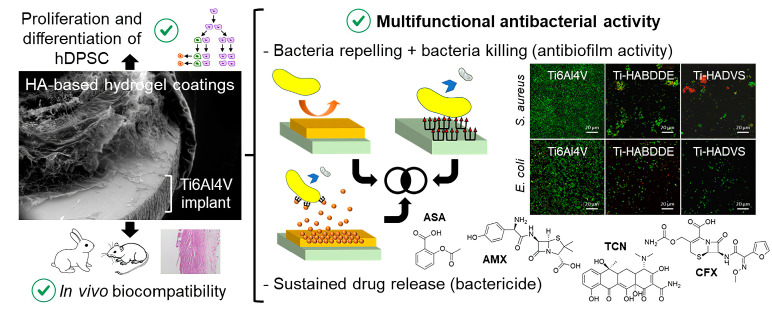
Graphical scheme and contact angle values. Reproduced with permission of [107]. Copyright 2023, Elsevier.

Each of the above three antibacterial methods has advantages and limitations. As mentioned above, dead microorganisms may accumulate after killing bacteria on the surface and affect the material properties. Releasing antibacterial agents also does not guarantee 100% elimination of bacteria; once bacteria are attached to the surface, they may multiply rapidly. To solve these limitations, we may combine two or more antibacterial mechanisms to prepare a hydrogel coating with excellent biocompatibility and antibacterial functions. For example, Jon et al. prepared HA-based hydrogel coatings on Ti6Al4V implants and used different crosslinking agents (1,4-butanediol diglycidyl ether, BDDE or divinyl sulfone, DVS) to modulate the physicochemical and nanomechanical properties of synthesized hydrogel coatings (Ti-HABDDE and Ti-HADVS) (Figure 12) [107]. Because the HA-based hydrogel coating has only excellent bacterial repellent function, in order to realize the bifunctional antibacterial mechanism (bacteria-repelling and bactericide-release), the researchers coated the antibacterial drug in the three-dimensional network structure of the hydrogel. Experiments showed that the coating had the ability to sustainedly release cefuroxime (CFX), tetracycline (TCN), amoxicillin (AMX, and acetylsalicylic acid (ASA). Relevant test data are as follows: HA-based hydrogel coatings demonstrated an outstanding multifunctional antibacterial activity: bacteria-repelling (51–55% of *S. aureus* and 27–40% of *E. coli*), bacteria-killing (82–119% of *S. aureus* and 83–87% of *E. coli*) and bactericide release killing (drug-loaded hydrogel coatings, R > 2).

## 4. Conclusions and Prospects

This paper provides a detailed review of the application of hydrogel coatings in biomedical antibacterial applications and introduces the principles of adhesion on the surface of materials and antibacterial strategies. Hydrogels can be attached to the surface of biomaterials in three ways: The first is surface-initiated graft crosslinking polymerization. The second is anchoring the hydrogel coating to the substrate surface. The third is the LbL self-assembly technique to coat crosslinked hydrogels. Hydrogel coatings’ antibacterial strategies are divided into three types: The first type is bacteria repellence and inhibition. The second type is the contact surface killing of bacteria. The third type is the release of antibacterial agents.

The antibacterial hydrogel coating interacts with organic and inorganic components as a biocompatible surface modifier, and the coating acts as a buffer between biomaterials and human tissues, making the biphasic interface of the material more stable and flexible and meeting the various needs of human tissue repair. The two key advantages of hydrogel coatings are as follows: Firstly, the coating can be firmly attached to the surface through chemical crosslinking and various anchoring reactions. Secondly, the coatings can attach to almost all kinds of materials, such as precious metals, oxides, polymers, and ceramics. Hydrogel coatings have excellent prospects for application, simple processing, stable performance, and wide application. Although significant progress has resulted from the research of antibacterial hydrogel coatings in biomedical applications, most of the research is only at the stage of cell and animal experiments, and further research on subsequent clinical applications needs to be conducted. The current research difficulties include the following: Firstly, the preparation method of antibacterial hydrogel coatings needs to be improved. Although the graft density of surface-initiated graft crosslinking polymerization is high, the initiator needs to be grafted to the surface, and the preparation process is relatively complex. The method of fixing the hydrogel coating to the substrate surface may result in uneven coverage of the hydrogel coating to the substrate surface due to the steric hinderance of the graft chain. Secondly, greater attention should be given to the study of the chemical stability of hydrogel coatings, including swelling, durability, degradability, mechanical properties, etc., which are important for the long-term effect of antibacterial hydrogel coatings on the human body. For example, swelling could be a problem for the coating of tubular medical devices, as the large swelling degree of hydrophilic hydrogels might block the tube. Finally, sterilization has been reported as an issue for most hydrogel coatings. In the complex environment of the body, the hydrogel coating needs to adapt to high temperature, high pressure, oxidation, and other conditions but also needs to maintain adhesion properties and bactericidal activity on the surface of the substrate. These problems will become the focus of future research on antibacterial hydrogel coatings. Future research directions may focus on the following aspects. First, there is a need to improve the adhesion of hydrogel coatings. The graft density should be large and uniform, so that the hydrogel coating uniformly covers the surface of the substrate, which is convenient for subsequent modification. Second, improvement of the mechanical properties of hydrogels and study of their long-term chemical stability are required, including improving the mechanical properties and breaking strength of hydrogels by crosslinking other chemicals. A third focus is the improvement of the swelling property of hydrogel, which is controlled by the change of pH and temperature. Improvement of this property is necessary for an intelligent and stable hydrogel coating. Fourth is the selection of materials that use monomers or segments with controllable degradation cycles and that have nontoxicity and harmless degradation products. This review provides a theoretical reference for follow-up research.

## Figures and Tables

**Table 1:** Comparison of advantages and disadvantages of different hydrogels in the biomedical field.

Hydrogel Type	Raw Material	Advantages	Disadvantages
Natural hydrogel	Collagen (protein)	Low antigenicity, Low inflammatory response, Excellent biological properties	High cost, high possibility of thrombosis, low mechanical strength, and difficult modification
	Gelatin (protein)	Low cost, Low immunogenicity, Biodegradable and biocompatible,	Poor stability at high temperature
	Silk fibroin (protein)	Excellent mechanical properties, Low immunogenicity, Blood clots less likely to form	Difficult source and slow gelling
	Glycosaminoglycan—Hyaluronic acid and Chondroitin sulfate (polysaccharide)	Mimics extracellular matrix components, Biodegradable, Binding cytokines	Degrades rapidly in vivo and requires crosslinking to stabilize
	Chitosan (polysaccharide)	Antibacterial, Low cost, Biocompatibility and biodegradability	Poor mechanical performance
Synthetic hydrogel	Polyethylene glycol (PEG)	Biodegradable, non-immunogenic	Lack of adhesive support
	Polyglutamic acid (PGA)	Biodegradable by hydrolysis, Thermoplastic, Mechanical properties are adjustable	Physical crosslinking is weak, hydrolytic products can induce inflammatory reaction and degrade rapidly
	Polylactic acid (PLA) and copolymer	Biodegradable by hydrolysis, Good mechanical properties It is soluble in organic solvents	Hydrolyzed byproducts can cause an inflammatory response

**Table 2:** Hydrogel coatings prepared by surface-initiated graft crosslinking polymerization.

First Author	Publication Year	Main Components of Hydrogel Coating	Loaded Substance	Coating Preparation Method	Coating Adhesion Principle	Crosslinking Agent	Function
Zhang [42]	2021	Acrylamide Acrylic acid	Ag nanoparticles/Antibiotics/Antimicrobial peptides	Photografted surfaces induce free radical polymerization	Dehydrogenation of benzophenone forms active free radicals on the main chain of the polyvinyl chloride polymer and induces monomer polymerization	UV crosslinking	Antithrombotic, antibacterial, effectively reduces platelet adhesion
Fu [43]	2021	HEMA, DMAPS, MMA	CS_2_	Ultraviolet photografting	Free radical polymerization	PEGDMA	Antibacterial and captures Ni^+^ through chelation of CS_2_
Wancura [46]	2020	PEGDA, APS, Functional gelatin	Protein	Redox mediated crosslinking technique	S_2_O_8_^2−^ and Fe^2+^ undergo redox reaction to form SO_4_^2−^ and free radicals and trigger free radical cross-linking		Multi-layer structure with different functional characteristics can be generated and the thickness can be controlled to enhance the cell adhesion function
Zhou [49]	2017	Polyethylene glycol dimethacrylate (PEGDMA)		Atom transfer radical polymerization (ATRP)		BiBB	Has good anti-biofilm and antibacterial effect against methicillin-resistant Staphylococcus aureus (MRSA)
Chen [50]	2021	GelGA, GelMA	Organic selenium	Apply and then light cure			Withstands balloon dilation, inhibits smooth muscle cell hyperproliferation, prevents thrombosis, and promotes NO production
Tugce [51]	2020	FuMaMAPEGMEMA	Biotin-benzyltetrazine	Rotating coating method (involving click chemistry)	Dopamine methyl acrylamide is anchored to the surface of titanium by catechol group, and methacrylate group is bonded to it by covalent bond	DMPA	Multifunctional hydrogels promote cell adhesion and proliferation
Liu [52]	2021	Poly (2-methylacryloxyethyl phosphate choline—dopamine methacrylate) (pMPCDA) copolymer		Mussel shell excitation chemical method	Michael addition between catechol and amino group, Schiff base addition and other reaction mechanisms		Anti-inflammatory and antithrombotic
Wu [56]	2021	Chitosan, gelatin	Ag^+^	3D printing technology	Silane coupling	Sodium Citrate	Promotes cell adhesion and bone growth

**Table 3:** Hydrogel coatings anchored to the surface of the substrate.

First Author	Publication Year	Main Components of Hydrogel Coating	Loaded Substance	Coating Preparation Method	Coating Adhesion Principle	Crosslinking Agent	Function
Leng Jin [65]	2021	GelMA, HAMA		UV crosslinking	ZnO layer anchored by catechol group on titanium surface	Photo-crosslinking	Reduce the toxicity of Zn^2+^, improve soft tissue compatibility and antibacterial ability
He Ye [66]	2021	GelMA	Photosensitizer Ce6-loaded polydopamine nanoparticles	After application, UV lamp crosslinking is performed	Anchored to the titanium surface by catecholic groups	Photo-crosslinking	Antibacterial, promotes cell adhesion and proliferation
Liu [67]	2021	Type A gelatin, AA, AAc-NHS ester, methacrylate anhydride monomer	Nano hydroxyapatite	Spin-coating method	Chemical bond anchoring	Ultraviolet light crosslinking	Promotes osteoblast differentiation and cell adhesion, and promotes wound healing

**Table 4:** Hydrogel coatings prepared by LbL self-assembly technique.

First Author	Publication Year	Main Components of Hydrogel Coating	Coating Preparation Method	Coating Adhesion Principle	Crosslinking Agent	Function
Cai [73]	2012	e-PEI, Alg	Alternate deposition method	Two-step annealing stabilizes the film onto the surface of the material	EDC	Antithrombotic, antibacterial
Wang [74]	2014	POEGDMAM, POEGMS	LbL thiol–ene “click” reactions	POEGDMAM first reacted with the thiols on the surface to form a single layer of polymer. Subsequently, POEGMS reacted with the immobilized ene groups on the polymer surface to give the second layer. Repeated deposition of the polymers gave the corresponding multilayer films.		Antibacterial

## Data Availability

Not applicable.
